# Extensively Drug-Resistant Carbapenemase-Producing *Pseudomonas aeruginosa* and Medical Tourism from the United States to Mexico, 2018–2019

**DOI:** 10.3201/eid2801.211880

**Published:** 2022-01

**Authors:** Ian Kracalik, D. Cal Ham, Gillian McAllister, Amanda R. Smith, Maureen Vowles, Kelly Kauber, Melba Zambrano, Gretchen Rodriguez, Kelley Garner, Kaitlyn Chorbi, P. Maureen Cassidy, Shannon McBee, Rhett J. Stoney, Kathleen Moser, Margarita E. Villarino, Oscar E. Zazueta, Amelia Bhatnagar, Erisa Sula, Richard A. Stanton, Allison C. Brown, Alison L. Halpin, Lauren Epstein, Maroya Spalding Walters, for the Verona Integron-Encoded Metallo-β-Lactamase–Producing Carbapenem-Resistant Pseudomonas aeruginosa Medical Tourism Investigation Team

**Affiliations:** Centers for Disease Control and Prevention, Atlanta, Georgia, USA (I. Kracalik, D. Cal Ham, G. McAllister, R.J. Stoney, K. Moser, M.E. Villarino, A. Bhatnagar, E. Sula, R.A. Stanton, A.C. Brown, A.L. Halpin, L. Epstein, M. Spalding Walters);; Utah Department of Health, Salt Lake City, Utah, USA (A.R. Smith, M. Vowles); Washington State Department of Health, Olympia, Washington, USA (K. Kauber);; Texas Department of State Health Services, Austin, Texas, USA (M. Zambrano, G. Rodriguez);; Arkansas Department of Health, Little Rock, Arkansas, USA (K. Garner);; Arizona Department of Health Services, Phoenix, Arizona, USA (K. Chorbi);; Oregon Health Authority, Portland, Oregon, USA (P.M. Cassidy);; West Virginia Department of Health and Human Resources, Charleston, West Virginia, USA (S. McBee);; Secretaría de Salud de Baja California, Mexicali, Mexico (O.E. Zazueta)

**Keywords:** outbreak, extensively drug-resistant, carbapenemase producing, Pseudomonas aeruginosa, bacteria, antimicrobial resistance, medical tourism, bariatric surgery, United States, Mexico

## Abstract

Carbapenem-resistant *Pseudomonas aeruginosa* (CRPA) producing the Verona integron‒encoded metallo-β-lactamase (VIM) are highly antimicrobial drug-resistant pathogens that are uncommon in the United States. We investigated the source of VIM-CRPA among US medical tourists who underwent bariatric surgery in Tijuana, Mexico. Cases were defined as isolation of VIM-CRPA or CRPA from a patient who had an elective invasive medical procedure in Mexico during January 2018‒December 2019 and within 45 days before specimen collection. Whole-genome sequencing of isolates was performed. Thirty-eight case-patients were identified in 18 states; 31 were operated on by surgeon 1, most frequently at facility A (27/31 patients). Whole-genome sequencing identified isolates linked to surgeon 1 were closely related and distinct from isolates linked to other surgeons in Tijuana. Facility A closed in March 2019. US patients and providers should acknowledge the risk for colonization or infection after medical tourism with highly drug-resistant pathogens uncommon in the United States.

In the United States, 20% of *Pseudomonas aeruginosa* from adult device‒associated healthcare-associated infections and 9% from surgical site infections are not susceptible to carbapenem antimicrobial drugs (*1*). However, only 1%–3% of carbapenem-resistant *P. aeruginosa* isolates harbor carbapenemases (*2*), enzymes typically encoded on mobile genetic elements that can be shared horizontally between bacteria and inactivate carbapenems and most other β-lactam antimicrobial drugs. These enzymes include active-on-imipenem (IMP) and V*erona integron*-encoded *metallo*-β-*lactamase (VIM). These* carbapenem-resistant *P. aeruginosa* (CP-CRPA) are associated with multidrug-resistant (MDR) phenotypes and can rapidly spread in healthcare settings because of poor infection prevention and control practices (*2*–*7*). Acquiring CP-CRPA is typically associated with receipt of healthcare; in the United States, cases have been linked to travel and domestic outbreaks (*8*,*9*). Carbapenemase-producing organisms might emerge in new geographic regions from inpatients who previously received healthcare in regions in which these organisms are more common (*10*–*12*).

VIM is the most commonly identified carbapenemase in *P. aeruginosa* worldwide (*13*). It is also the most common carbapenemase identified in *P. aeruginosa* in the United States, although the absolute number of cases remains low. During 2017–2018, ≈200 VIM-CRPA were identified among nearly 15,000 isolates tested nationally (https://arpsp.cdc.gov/profile/arln/crpa).

Annually, up to 750,000 US residents participate in medical tourism, defined as international travel for the purpose of receiving medical care (*14*,*15*). Motivations for medical tourism often include lower cost, shorter wait times, and fewer medical requirements (*15*,*16*). Among medical tourists surveyed in 11 US states and territories during 2016, Mexico was the most common destination country (*16*). The exact number of US medical tourists who undergo bariatric surgery annually is unknown, but in a 2017 survey, 10 Mexico-based bariatric surgeons reported performing >2,500 procedures on medical tourists, most of whom were US residents (*17*). One study estimated 2% of bariatric surgeries worldwide are performed on medical tourists; most of them were performed in Mexico (*17*).

Several infectious disease outbreaks linked to medical tourism have been reported, including nontuberculous mycobacteria surgical site infections among medical tourists from the United States and Switzerland undergoing cosmetic surgery in Latin America (*18*,*19*) and Q fever among US medical tourists receiving live cell therapy in Germany (*20*). In this report, we describe an outbreak of extensively drug-resistant (XDR) *P. aeruginosa* harboring *bla*_VIM_ (VIM-CRPA) among US medical tourists who underwent bariatric surgery in Tijuana, Mexico, during 2018–2019.

## Methods

### Outbreak Identification and Early Epidemiologic Investigation

On September 28, 2018, the Centers for Disease Control and Prevention (CDC; Atlanta, GA, USA) received a report from the Arizona Department of Health Services of VIM-CRPA cultured from an abdominal wound of a 31-year-old patient on September 5, 2018. Initial investigation determined the patient underwent bariatric surgery in Tijuana, Mexico, 15 days before specimen collection. From late September through late November 2018, CDC received 6 reports of VIM-CRPA isolates from patients who underwent bariatric surgery in Tijuana. Four patients used the same US-based travel agency (travel agency A), which coordinated travel and arranged care for medical tourists; all 4 patients reported undergoing bariatric surgery at the same facility in Tijuana (facility A) with the same surgeon (surgeon 1).

In response, CDC and the Secretariat of Health in Baja California, Mexico, launched a public health investigation. On November 19, 2018, CDC issued a call for cases on the Epidemic Information Exchange (https://www.emergency.cdc.gov/epix/index.asp) for *P. aeruginosa* isolated from patients reporting bariatric surgery in Tijuana since August 1, 2018; CDC also posted an Emerging Infections Network notification on November 23, 2018. The Federal Commission for Protection against Sanitary Risk in Mexico conducted an infection control assessment of facility A in December 2018 and identified multiple lapses, including poor hand hygiene practices, incomplete clinical records, and lack of chemical or biologic indicators to ensure medical equipment and device sterility after reprocessing. The lack of indicators potentially exposed patients to risk for infections with bloodborne pathogens, such as HIV and hepatitis B and C viruses, in addition to bacterial infections. On the basis of these findings, the Secretariat of Health issued a closure order for the surgical suite at facility A on December 17, 2018, and CDC issued an Alert Level 2 Travel Health Notice during January 2019, advising US residents against undergoing surgery at Facility A (Appendix).

### Case Definition

A confirmed case was isolation of VIM-CRPA from a patient who had an elective invasive medical procedure in Mexico during January 2018–December 2019 and within 45 days before specimen collection. A probable case was isolation of CRPA, with the isolate unavailable for carbapenemase testing, from a patient who had an elective invasive medical procedure in Mexico during January 2018–December 2019 and within 45 days before specimen collection. A suspect case was infection (subjective or measured fever and >2 of the following at incision sites: pus; fluid draining; or warmth, redness and swelling) within 45 days of surgery in a patient who had surgery at facility A during January 2018–December 2019 and sought medical care but did not have a culture collected.

### Passive Case Finding

CRPA are routinely submitted from clinical laboratories to the Antibiotic Resistance Laboratory Network, a US national network of 55 public health laboratories performing carbapenemase testing for carbapenem-resistant organisms. There is no national requirement to report or submit CRPA for carbapenem resistance mechanism testing, and isolate submission strategies differ by state. CDC guidance for containing spread of emerging and targeted MDR organisms recommends state and local health departments investigate reports of novel or targeted carbapenemase-producing organisms, including CP-CRPA (*21*). After the initial cluster was identified, health departments investigating cases in persons who reported surgery in Tijuana attempted to obtain the names of healthcare facilities, surgeons, and travel agencies used by case-patients; the type of surgery performed; and whether the case-patient was subsequently admitted to a US healthcare facility. During some case investigations, case-patients reported knowing other sick persons who underwent surgery; state and local health departments attempted to contact these persons and review medical records for those who reported signs or symptoms of infection.

### Patient Notification and Active Case Finding

Because names of persons who underwent surgery at facility A were not initially available, in January 2019, CDC posted an online notification for patients and their US healthcare providers (https://www.cdc.gov/hai/outbreaks/pseudomonas-aeruginosa.html) and an Alert Level 2 Travel Health Notice (Appendix), both of which were covered by major media outlets (*22*–*24*). Notifications provided warning of postoperative bacterial infection risk and potential for bloodborne pathogen transmission. Individual states also issued Health Alert Network notices to increase awareness of potential cases. During March 2019, travel agency A sent an electronic notification regarding potential exposures to clients who had surgery at facility A during August 1, 2018–February 15, 2019, and provided CDC with contact information for persons referred to facility A during August 1, 2018–March 1, 2019.

We classified persons who had surgery on or after January 1, 2019, as higher risk for new onset or ongoing postoperative infections; persons who had surgery before January 1, 2019, were classified as lower risk because of the longer elapsed time since surgery. For higher risk persons, CDC and state and local health departments conducted telephone notifications and structured interviews to obtain demographics, clinical and exposure details, and information about factors influencing their decision to have surgery at facility A (Appendix). In addition to the travel agency A client notification and CDC and online notification, CDC recommended state and local public health officials send notification letters to lower risk persons; some health jurisdictions additionally performed active outreach for these persons. Contact information for non-US residents was shared with respective public health agencies. For case-patients admitted to US healthcare facilities, responses were conducted by health departments to assess for transmission (https://www.cdc.gov/hai/containment/guidelines.html).

### Molecular Typing and Antimicrobial Drug Susceptibility Testing

VIM-CRPA isolates underwent whole-genome sequencing (WGS) and analysis at CDC and state health departments. WGS libraries were prepared by using the NuGEN Ovation Ultralow System V2 Assay Kit (NuGen Technologies, https://www.nugentechnologies.co.za) and sequenced by using the MiSeq Reagent Kit v2 (500 cycle) (https://www.illumina.com) and the MiSeq System (Illumina), generating 2 × 250 paired-end reads. CDC processed and analyzed all sequences by using bioinformatics pipeline QuAISAR-H (quality, assembly, species identification, sequence typing, annotation, resistance mechanisms for healthcare pathogens) (https://github.com/DHQP/QuAISAR_singularity) and assessed phylogeny by using a core genome multilocus sequence type scheme for *P. aeruginosa* and SNVPhyl (*25*–*27*).

Antimicrobial susceptibility testing for 15 drugs was performed at CDC by using frozen broth microdilution panels prepared according to Clinical and Laboratory Standards Institute reference methods (*28*). MICs were interpreted as susceptible, intermediate, or resistant according to Clinical and Laboratory Standards Institute definitions (*29*). We classified isolates as MDR or XDR by using published definitions (*30*).

### Statistical Analysis

We analyzed epidemiologic data by using R statistical software version 3.5.2 (R Foundation for Statistical Computing, https://cran.r-project.org). We estimated the VIM-CRPA attack rate by using data from the higher risk group (patients who had surgery during January–March 2019) with confirmed and probable cases from clinical cultures from patients who underwent surgery at facility A as the numerator and total patients referred by travel agency A to facility A as the denominator. We limited epidemiologic analyses to probable and confirmed cases.

### Ethics

This project was reviewed by human subjects advisors in the National Center for Emerging and Zoonotic Infectious Diseases at CDC and received a nonresearch determination and emergency approval by the Office of Management and Budget (OMB Control No. 0920–1253). This activity was reviewed by CDC and was conducted consistent with applicable federal law and CDC policy. All patients who participated in the structured interviews provided informed consent.

## Results

### Epidemiologic Investigation

**Figure 1:**
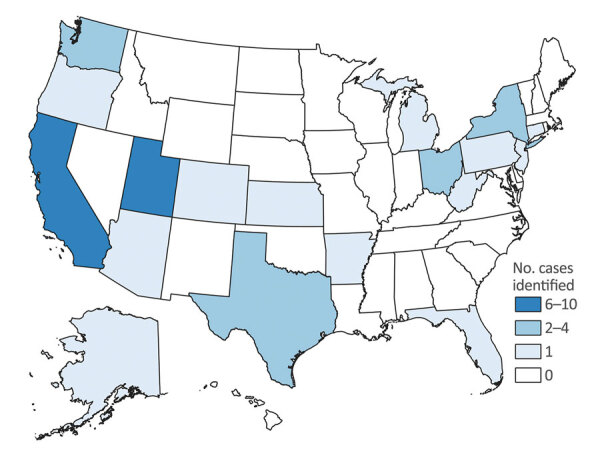
Confirmed and probable cases of infection with Verona integron‒encoded, metallo-β-lactamase‒producing, carbapenem-resistant *Pseudomonas aeruginosa*, by state in which bacterium was identified, among US medical tourists undergoing elective invasive procedures in Tijuana, Mexico, January 2018–December 2019. Six suspected cases, from Arizona (n = 1), Georgia (n = 3), Michigan (n = 1), and Washington (n = 1) are not shown.

During August 1, 2018–December 31, 2019, we identified 44 cases from 19 states; 25 were confirmed cases, 13 were probable cases, and 6 were suspected cases (Figure 1). Among the 38 patients who had confirmed or probable cases, 34 (89.5%) were female, and the median age at time of specimen collection was 39 (interquartile range 31–48) years (Table 1). Sleeve gastrectomy was the most common surgical procedure, reported by 34 (89.5%) of 38 case-patients. Median time from surgery to specimen collection was 12 (range 3–40) days. After surgery in Tijuana, 16 (42.1%) of 38 case-patients were hospitalized in the United States. Among the 14 hospitalized patients for which the duration of hospitalization was known, the median stay was 7 (range 1–19) days.

**Table 1:** Characteristics for 38 confirmed and probable case-patients who had Verona integron‒encoded, metallo-β-lactamase‒producing *Pseudomonas aeruginosa* among US medical tourists traveling to Tijuana, Mexico, January 2018–December 2019

Characteristic	No. (%)
Age, y	
23–34	14 (37)
35–49	15 (40)
50–64	6 (16)
≥65	1 (3)
Unknown	2 (5)
Sex	
F	34 (90)
M	4 (10)
Surgical facility	
Facility A	27 (71)
Facility B	1 (3)
Facility C	1 (3)
Facility D	1 (3)
Facility E*	2 (5)
Facility F	1 (3)
Facility G	1 (3)
Facility H	1 (3)
Facility I	2 (5)
Facility J	1 (3)
Unknown	1 (3)
Surgeon who performed procedure	
Surgeon 1	31 (82)
Surgeon 2	1 (3)
Surgeon 3	1 (3)
Surgeon 4	1 (3)
Surgeon 5	1 (3)
Unknown	3 (8)
Surgical procedure†	
Sleeve gastrectomy	34 (90)
Cholecystectomy	1 (3)
Laparoscopic adjustable gastric band	1 (3)
Sleeve gastrectomy revision	1 (3)
Unspecified bariatric surgery	3 (8)
Specimen source	
Wound	31 (82)
Intraabdominal abscess	4 (11)
Abdominal fluid	1 (3)
Blood	1 (3)
Rectal swab specimen	1 (3)
Hospitalized in the United States after surgery	16 (42)
Patient died within 30 d of specimen collection	1 (3)

*Patient reported exposure to facilities E and D. †Four patients underwent >1 procedure: gastrectomy and cholecystectomy (n = 1), sleeve gastrectomy and bowel resection (1), sleeve gastrectomy and lap band removal (1), and sleeve gastrectomy, breast augmentation, and abdominoplasty (1).

Four hospitalized case-patients were admitted to the intensive care unit; for 8 case-patients, this admission status was unknown. Six hospitalized case-patients underwent surgery because of their infection; for 5, surgery for postsurgical infection management status was unknown. One of the 16 hospitalized case-patients died in the in the hospital 9 days after sleeve gastrectomy surgery. For this patient, who underwent a procedure at facility E by surgeon 1, VIM-CRPA was identified from a screening rectal swab specimen at admission. Whether the patient had VIM-CRPA infection or the surgery at facility E otherwise contributed to death is unclear from available medical records. All other case-patients had VIM-CRPA infections on the basis of results from clinical cultures. From our investigation, no evidence of onward transmission in the US healthcare facilities in which case-patients were hospitalized was identified.

For the confirmed and probable cases, 37 (97%) case-patients named 10 Tijuana facilities in which they underwent invasive procedures. Most reported surgery at facility A (27/38; 71.1%) (Table 1). Among the 35 case-patients who reported the name of their surgeon, 31 (88.6%) named surgeon 1, including the 27 case-patients who underwent surgery at facility A and 4 case-patients who underwent surgery at other or unknown facilities.

**Figure 2:**
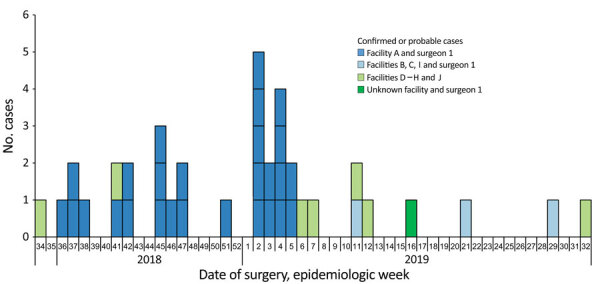
Confirmed and probable cases of infection with Verona integron‒encoded, metallo-β-lactamase‒producing, carbapenem-resistant *Pseudomonas aeruginosa*, by week of surgery, among US medical tourists undergoing elective invasive procedures in Tijuana, Mexico, January 2018‒December 2019. Dark blue bars show cases associated with surgery performed at facility A by surgeon 1; light green bars show cases associated with surgery at facilities D–H and J by surgeons other than surgeon 1; and light blue bars show cases associated with facilities B, C, and I by surgeon 1; and dark green bar shows a case associated with surgeon 1 and an unknown facility. A confirmed case was isolation of Verona integron‒encoded, metallo-β-lactamase‒producing, carbapenem-resistant *P. aeruginosa* from a patient who had an elective invasive medical procedure in Mexico during January 2018–December 2019 and within 45 days before specimen collection. A probable case was isolation of carbapenem-resistant *P. aeruginosa*, with an isolate unavailable for carbapenemase testing, from a patient who had an elective invasive medical procedure in Mexico during January 2018–December 2019 and within 45-days before specimen collection. No cases were identified from patients who underwent surgery before August 2018 (week 34). The peak of the outbreak encompassed epidemiologic weeks 2–5 (January 2019).

Surgery dates ranged from August 2018 to August 2019 (Figure 2). Confirmed and probable cases in patients who underwent surgery at facility A peaked during January 2019 (epidemiologic weeks 2–5); 13 (48.2%) of 27 case-patients who reported surgery at facility A had a procedure during this 4-week period when the surgical suite was reported closed. Facility A closed permanently in early March 2019; 4 case-patients associated with surgeon 1 had surgery after this date at facility I (n = 2 case-patients), facility E, and an unknown facility in the Tijuana area. Ongoing monitoring through December 2019 did not identify additional cases linked to facility A after January 2019 (epidemiologic week 5) or to surgeon 1 after July 2019 (epidemiologic week 29).

### Patient Notification and Active Case Finding

During August 1, 2018–March 1, 2019, travel agency A referred 793 persons from 6 countries for surgery at facility A; of these persons, 743 (94%) were US residents. Health authorities in the other countries were contacted to inform them of the outbreak, and we were not notified of any cases. We interviewed 160 (21%) US residents who underwent surgery, including 92 (46%) of 200 persons in the higher risk group targeted for active outreach and 68 (13%) of 543 persons in the lower risk group and for whom some health jurisdictions performed active outreach. Fifteen cases were identified through interviews. Overall, passive and active case finding identified 7 confirmed case-patients and 6 probable case-patients who underwent surgery at facility A and who were among the 200 persons in the higher risk group; therefore, the attack rate for VIM-CRPA for persons who had surgery at facility A during January–March 2019 was 13/200 (6.5%, 95% CI 3.6%‒10.8%).

Interviewed persons who were not confirmed or probable case-patients (n = 148) were demographically similar to confirmed and probable case-patients (n = 38). The most common reason for undergoing surgery abroad was lower cost, reported by 132 (82.5%) of the 160 interviewed persons. Among the 41 persons who reported being aware of the CDC travel advisories or negative media stories before their surgery, cost was the most common reason for proceeding with surgery (n = 22, 53.7%) (Table 2).

**Table 2:** Characteristics and interview responses from 160 US residents who underwent surgery at facility A in Tijuana, Mexico, August 1, 2018–March 1, 2019*

Characteristic	Total, n = 160				Case status‡		
		Risk period†			Confirmed, probable, and suspected, n = 18	Confirmed and probable, n = 12	Noncases, n = 142
		High risk, n = 92	Low risk, n = 68				
Sex							
M	28 (18)	20 (22)	8 (12)		2 (11)	0	26 (18)
F	132 (83)	72 (78)	60 (88)		16 (89)	12 (100)	116 (82)
Median age y (IQR)	43 (34–49)	43 (36–49)	43.5 (32–49)		45 (32.5–52.5)	41 (31–53)	43 (34.3–49)
Unknown or declined to answer	11 (7)	7 (8)	4 (6)		1 (6)	1 (8)	10 (7)
Procedure performed							
Gastric sleeve	131 (82)	70 (76)§	61 (90)§		15 (83)	10 (83)	116 (82)
Laparoscopic gastric sleeve¶	50 (31)	23 (25)§	27 (40)§		5(28)	4 (33)	45 (32)
Unspecified gastric sleeve#	79 (49)	47 (51)	32 (47)		10 (56)	6 (50)	69 (49)
Open gastric sleeve	2 (1)	0	2 (3)		0	0	2 (1)
Revision of previous surgery		8 (9)	2 (3)		3 (17)	3 (25)**	7 (5)
Gastric bypass	9 (6)	5 (5)	4 (6)		0	0	9 (6)
Other	9 (6)	4 (4)	5 (7)		0	0	9 (6)
Unknown or declined to answer	11 (7)	10 (11)§	1 (2)§		1 (6)	0	10 (7)
Surgeon who performed procedures							
Surgeon 1	139 (87)	82 (89)	57 (84)		16 (89)	11 (92)	123 (87)
Surgeon 6††	1 (<1)	1 (1)	0		0	0	1 (<1)
Unknown or declined to answer	20 (13)	9 (10)	11 (16)		2 (11)	1 (8)	18 (13)
Factors contributing to decision to have surgery abroad							
Lower cost	132 (83)	75 (82)	57 (84)		14 (78)	9 (75)	118 (83)
Recommendations from friends or family	83 (52)	46 (50)	37 (54)		8 (44)	7 (58)	75 (53)
Did not meet weight qualifications for surgery in the United States	36 (23)	22 (24)	14 (21)		1 (6)	1 (8)	35 (25)
Convenience/shorter wait time	36 (23)	22 (24)	14 (21)		7 (39)	6 (50)**	29 (20)
Insurance would not approve/lack of insurance	9 (6)	3 (3)	6 (9)		1 (6)	1 (8)	8 (6)
Other	13 (8)	8 (9)	5 (7)		3 (17)	0	10 (7)
Unknown or declined to answer	7 (4)	5 (5)	2 (3)		0	0	7 (5)
Had concerns over quality of care before surgery (yes), n = 151	45 (30)	30 (36)	15 (22)		7 (41)	4 (36)	38 (28)
Of those with concerns, what were they?, n = 45							
Surgery in a foreign country	13 (29)	8 (27)	5 (33)		3 (43)	2 (50)	10 (26)
Infection or other adverse outcome	8 (18)	7 (23)	1 (7)		3 (43)	2 (50)	5 (13)
General	11 (24)	6 (20)	5 (33)		0	0	11 (29)
Unknown or declined to answer	14 (31)	10 (33)	4 (27)		1 (14)	0	13 (34)
Aware of negative media stories or travel advisories about facility A before surgery (yes), n = 155‡‡	41 (27)	24 (27)	17 (25)		7 (39)	6 (50)	34 (25)
Awareness from CDC Travel Health Notice before surgery, n = 41§§	5 (12)	5 (21)	0		3 (43)**	3 (50)**	2 (6)
Factors contributing to decision to have surgery, n = 41							
Cost	22 (54)	12 (50)	10 (59)		4 (57)	3 (50)	18 (53)
Already paid	9 (22)	8 (33)	1 (6)		2 (29)	1 (17)	7 (21)
Unable to receive deposit refund	3 (7)	2 (8)	1 (6)		1 (14)	0	2 (6)
Already booked travel	6 (15)	6 (25)^‡^	0^‡^		2 (29)	1 (17)	4 (12)
Short wait time	2 (5)	2 (8)	0		1 (14)	0	1 (3)
Received assurances from travel agency/facility	16 (39)	12 (50)	4 (24)		5 (71)	4 (67)	11 (32)
Received assurances from previous patients	9 (22)	6 (25)	3 (18)		2 (29)	1 (17)	7 (21)
Other	8 (20)	2 (8)§	6 (35)§		2 (29)	1 (17)	6 (18)
Unknown or declined to answer	1 (2)	1 (4)	0		0	0	1 (3)

*Values are no.(%) unless indicated otherwise. CDC, Centers for Disease Control and Prevention; IQR, interquartile range. †Persons who had surgery on or after January 1, 2019, were classified as higher risk for new onset or ongoing postoperative infections. Persons who had surgery before January 1, 2019, were classified as lower risk because of the longer elapsed time since surgery. ‡A confirmed case was isolation of Verona integron‒encoded, metallo-β-lactamase‒producing, carbapenem-resistant *Pseudomonas aeruginosa* from a patient who had surgery during January 2018–December 2019, and within 45 d before specimen collection. A probable case was isolation of carbapenem-resistant *P. aeruginosa*, with isolate unavailable for carbapenemase testing, from a patient who had surgery during January 2018–December 2019, and within 45 d before specimen collection. A suspected case was infection (subjective or measured fever and >2 of the following at incision sites: pus, fluid draining, or warmth, redness and swelling) within 45 d of surgery in a patient who had surgery during January 2018–December 2019, and sought medical care but did not have a culture collected. §Statistically significant difference; p<0.05. ¶One patient underwent laparoscopic gastric sleeve and cholecystectomy. #Four patients underwent unspecified gastric sleeve and cholecystectomy. **Statistically significant difference compared with noncases; p<0.05. ††Persons who were interviewed reported surgery by surgeon 1 and another surgeon (surgeon 6) not reported by any case-patients. ‡‡Negative media stories about Facility A might have predated this outbreak §§Centers for Disease Control and Prevention issued an Alert Level 2 Travel Health Notice on January 9, 2019.

### Microbiologic Investigation

**Figure 3:**
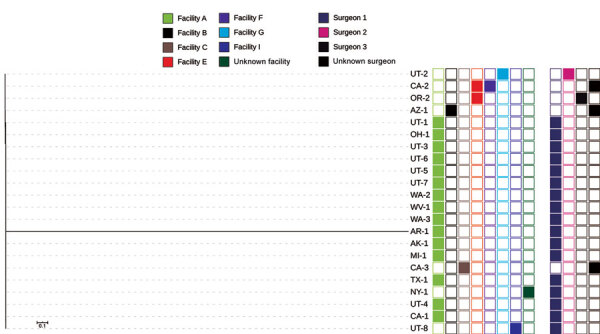
Whole-genome sequencing analysis and selected epidemiologic data for 22 Verona-integron-encoded metallo-β-lactamase-producing carbapenem-resistant *Pseudomonas aeruginosa* clinical isolates from US medical tourists who underwent surgery in Tijuana, Mexico, August 2018–December 2019. Phylogenetic tree includes an outlier isolate from Arkansas. On the right, the first group of 8 columns indicates facilities (A, B,C, E, F, G, I, and unknown), and the second group of 4 columns indicates surgeons (1, 2, 3, and unknown). Scale bar indicates nucleotide substitutions per site.

**Figure 4:**
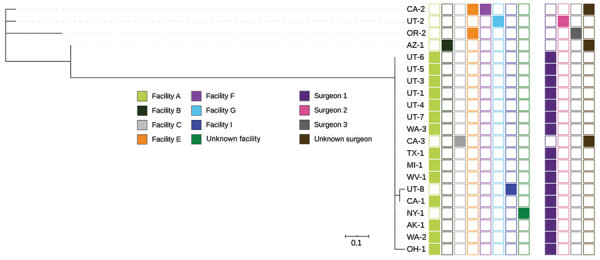
Whole-genome sequencing analysis and selected epidemiologic data for 21 Verona-integron-encoded metallo-β-lactamase-producing carbapenem-resistant *Pseudomonas aeruginosa* clinical isolates from US medical tourists who underwent surgery in Tijuana, Mexico, August 2018–December 2019. Phylogenetic tree excludes an outlier isolate from Arkansas. On the right, the first group of 8 columns indicates facilities (A, B,C, E, F, G, I, and unknown), and the second group of 4 columns indicates surgeons (1, 2, 3, and unknown). Scale bar indicates nucleotide substitutions per site.

Isolates from 22 of 25 confirmed cases underwent WGS. All isolates harbored *bla*_VIM-2_; 21 were sequence type (ST) 111 and 1 was a novel ST. Overall, isolates varied by 0 to 4,375 single nucleotide variants (SNVs) over a 90.08% core genome (Figures 3, 4). Seventeen isolates formed a distinct cluster varying by 0 to 4 SNVs over a 93.29% core genome and were associated with surgeon 1 (n = 16) or an unknown surgeon (n = 1) and with >3 different facilities. One isolate associated with surgeon 1 was the novel ST and differed by 4,375 SNVs, indicating that it was not closely related to other isolates associated with facility A (Figure 3). The remaining 4 isolates differed by 5‒18 SNVs over an 96.34% core genome; among these, 2 were associated with facility E but were not more closely related to each other than to isolates associated with facilities B and G. Antimicrobial susceptibility testing was performed for 10 isolates at the request of health departments to guide treatment. All isolates were XDR and resistant to ceftazidime/avibactam and ceftolozane/tazobactam (Table 3).

**Table 3:** Susceptibility of 10 Verona integron-encoded, metallo-β-lactamase‒producing, carbapenem-resistant *Pseudomonas aeruginosa* isolates from US medical tourists traveling to Tijuana, Mexico, January 2018–December 2019*

ID no.	MIC, μg/mL														
	AMK	ATM	FEP	CAZ	CZA	C/T	CIP	CST	DOR	GEN	IPM	LVX	MEM	TZP	TOB
15	64 (R)	16 (I)	16 (I)	32 (R)	>16/4 (R)	>16/4 (R)	>8 (R)	1 (S)	>8 (R)	4 (S)	>64 (R)	>8 (R)	>8 (R)	32/4 (I)	>16 (R)
14	16 (S)	32 (R)	32 (R)	32 (R)	>16/4 (R)	>16/4 (R)	>8 (R)	1 (S)	4 (I)	16 (R)	>64 (R)	>8 (R)	8 (R)	64/4 (I)	>16 (R)
22	64 (R)	16 (I)	16 (I)	32 (R)	>16/4 (R)	>16/4 (R)	>8 (R)	1 (S)	>8 (R)	4 (S)	>64 (R)	>8 (R)	>8 (R)	64/4 (I)	>16 (R)
1	64 (R)	16 (I)	16 (I)	32 (R)	>16/4 (R)	>16/4 (R)	>8 (R)	1 (S)	>8 (R)	4 (S)	>64 (R)	>8 (R)	>8 (R)	64/4 (I)	>16 (R)
3	64 (R)	16 (I)	16 (I)	32 (R)	>16/4 (R)	>16/4 (R)	>8 (R)	2 (S)	>8 (R)	2 (S)	>64 (R)	>8 (R)	>8 (R)	64/4 (I)	>16 (R)
5	64 (R)	16 (I)	16 (I)	32 (R)	>16/4 (R)	>16/4 (R)	>8 (R)	1 (S)	>8 (R)	2 (S)	>64 (R)	>8 (R)	>8 (R)	32/4 (I)	>16 (R)
9	32 (I)	16 (I)	16 (I)	32 (R)	>16/4 (R)	>16/4 (R)	>8 (R)	2 (S)	>8 (R)	2 (S)	>64 (R)	>8 (R)	>8 (R)	32/4 (I)	>16 (R)
8	32 (I)	16 (I)	16 (I)	32 (R)	>16/4 (R)	>16/4 (R)	>8 (R)	2 (S)	>8 (R)	2 (S)	>64 (R)	>8 (R)	>8 (R)	32/4 (I)	>16 (R)
10	32 (I)	16 (I)	16 (I)	32 (R)	>16/4 (R)	>16/4 (R)	>8 (R)	2 (S)	>8 (R)	2 (S)	>64 (R)	>8 (R)	>8 (R)	32/4 (I)	>16 (R)
13	32 (I)	16 (I)	16 (I)	32 (R)	>16/4 (R)	>16/4 (R)	>8 (R)	2 (S)	>8 (R)	4 (S)	>64 (R)	>8 (R)	>8 (R)	32/4 (I)	>16 (R)

*Isolates were tested against 15 antimicrobial drugs by using reference broth microdilution. All isolates were ST111, except for 14 which was a unique ST. AMK, amikacin; ATM, aztreonam; C/T, ceftolozane/tazobactam; CAZ, ceftazidime; CIP, ciprofloxacin; CST, colistin; CZA, ceftazidime/avibactam; DOR, doripenem; FEP, cefepime; GEN, gentamicin; I, intermediate; ID, identification; IPM, imipenem; LVX, levofloxacin; MEM, meropenem; R, resistant; S, sensitive; ST, sequence; TOB, tobramycin; TZP, piperacillin/tazobactam.

## Discussion

We describe a large, prolonged outbreak of XDR VIM-CRPA among US medical tourists who underwent bariatric surgery in Tijuana, Mexico. Most isolates were clonal and linked to a surgeon who operated at multiple healthcare facilities; we also identified isolates genetically distinct from this outbreak strain and associated with other healthcare facilities and surgeons. Although serious complications from laparoscopic sleeve gastrectomy in the United States are uncommon (≈1%–2%) (*31*,*32*), >40% of case-patients in our investigation required postoperative hospitalization in the United States, highlighting the severity of infections. Active outreach to exposed persons accounted for one third of case-patients identified. Our investigation underscores the potential for medical tourism to introduce highly concerning pathogens into the US healthcare system.

Several lines of epidemiologic and laboratory findings in this investigation support a point source outbreak linked to surgeon 1, a surgeon specializing in bariatric surgery who operated primarily at facility A, although the exact source of pathogen was not identified. VIM-CRPA infections appeared to increase starting in September 2018 and decreased in March 2019 after travel agency A notified exposed clients and stopped referrals to facility A. Clonal strains isolated from case-patients after surgery performed by surgeon 1 across multiple facilities, and infection control lapses at facility A led us to hypothesize a persistently contaminated mobile medical device; a laparoscope transported between facilities with surgeon 1 was a plausible outbreak source. *P. aeruginosa* is known to persistently colonize medical devices, including flexible endoscopes (*33*,*34*); in Brazil, surgeons transporting their own laparoscopic equipment between different hospitals were the suspected source of a multifacility *Mycobacterium* spp. outbreak (*35*). Alternative explanations include a widespread persistently contaminated environmental or water source at facility A or a persistently colonized healthcare worker, such as surgeon 1.

We also identified case-patients with VIM-CRPA who were not epidemiologically linked to facility A or surgeon 1 and isolates that were genetically distinct from the outbreak cluster. These infections appeared to be sporadic. Although 2 of these case-patients underwent surgery at the same facility, their isolates were not more closely related to each other than to those from case-patients who underwent surgery at other facilities, decreasing suspicion for a second outbreak. Similar to most isolates linked to procedures performed by surgeon 1, these sporadic cases belonged to ST111, which has been associated with epidemic spread of carbapenemases in *P. aeruginosa* globally (*36*). Since July 2020, CDC has received 6 additional reports of VIM-CRPA cases among US residents who had undergone elective invasive medical procedures in Tijuana, none of whom were reported to have a common procedure, facility or surgeon; however, 1 case-patient was operated on by surgeon 1. These recent infections underscore the potential for US residents to acquire highly resistant bacteria when receiving medical care abroad, even in the absence of a recognized outbreak. In some countries, MDR organisms rarely identified in the United States may be more common, increasing the potential for acquiring resistant organisms, regardless of quality of care. Persons considering medical tourism and US healthcare providers caring for prospective or returned medical tourists should be aware that standards for infection control, as well as regulations and enforcement practices, vary by country and facility (*37*). US public health authorities and healthcare providers might have limited access to information to inform recommendations for follow-up care or testing for medical tourists.

In the United States, carbapenemases are rarely the cause of carbapenem resistance in *P. aeruginosa*, and few clinical laboratories perform carbapenemase testing for CRPA. Despite increased carbapenemase testing for CRPA through the Antibiotic Resistance Laboratory Network, our investigation shows CP-CRPA continues to be underdetected. Nearly 1 in 3 cases in this investigation represent CRPA clinical isolates that were not tested for carbapenemases, despite being highly resistant and identified from patients who had medical histories of concern during a well-publicized outbreak (*22*). CP-CRPA are overwhelmingly MDR and often XDR (*38*). Identification of these antimicrobial susceptibility testing phenotypes, especially in patients with a history of healthcare outside the United States, should increase suspicion for CP-CRPA.

Despite warnings from US public health agencies, medical tourists continued to undergo surgery at facility A during January 1–March 1, 2019. Nearly 30% of interviewed persons who had surgery were aware of the outbreak or negative news stories associated with facility A before their surgery; however, interviewed persons might have made travel or surgery reservations and deposits before issuance of travel health notices, which could have influenced their decisions to proceed with the procedure. Consistent with a 2015 survey of bariatric medical tourists from Canada, we found primary motivations for bariatric medical tourism among interviewees included shorter wait times and lower cost (*39*). A qualitative study from Canada also showed that bariatric medical tourists identified the Internet as a primary source of information for identifying providers and validating decisions to engage in medical tourism (*40*). Difficulty reconciling conflicting information sources might have delayed the effect of the CDC travel warnings.

Our investigation had several limitations. Because of limited data from the outbreak facility, its international setting, and lack of environmental cultures, we could not determine the outbreak source, although several hypotheses were considered. Additional cases might have gone undetected for 2 reasons. First, CP-CRPA is underdetected because of low suspicion of the potential for CRPA to harbor carbapenemases and limited availability of testing for carbapenemases. Second, active outreach was limited in several ways: only referrals from travel agency A, rather than all surgical patients at facility A, were available to US public health authorities; we focused efforts on persons who were at greatest risk for having current or new-onset infections, but <50% of targeted persons were reached. Because of high nonresponse rates and underdetection of CP-CRPA, our calculated attack rate during January–March 2019 is probably a lower bound; however, additional patients could have undergone surgery who were not included on our list, thereby overestimating the attack rate. Third, although transmission to household contacts of case-patients was not identified, this transmission was not routinely assessed for all case-patients. Fourth, persons interviewed might have been more likely to have infections compared with other facility A patients and possibly differed in their motivations for medical tourism and awareness of public health notifications, and might not be representative of all facility A patients or Tijuana bariatric medical tourists.

In this investigation, epidemiologic and molecular data link a single surgeon, performing surgeries at multiple facilities, to a prolonged outbreak among medical tourists. US patients and providers should be aware of the risk for colonization and infection with highly resistant pathogens not commonly encountered in the United States after medical tourism.

Appendix Additional information on extensively drug-resistant carbapenemase-producing *Pseudomonas aeruginosa* and medical tourism from United States to Mexico, 2018–2019.
