# Supplementary material for: Extensively Drug-Resistant Carbapenemase-Producing Pseudomonas aeruginosa and Medical Tourism from the United States to Mexico, 2018–2019
Source: Emerg Infect Dis. 2022 Jan;28(1):51–61. doi: 10.3201/eid2801.211880 (PMC8714193; doi:10.3201/eid2801.211880)
Supplement: Appendix — Additional information on extensively drug-resistant carbapenemase-producing Pseudomonas aeruginosa and medical tourism from United States to Mexico, 2018–2019. [file 21-1880-Techapp-s1.pdf]

Article DOI: https://doi.org//10.3201/eid2801.211880

Extensively Drug-Resistant Carbapenemase-Producing *Pseudomonas aeruginosa* and Medical Tourism from United States to Mexico, 2018–2019

Appendix

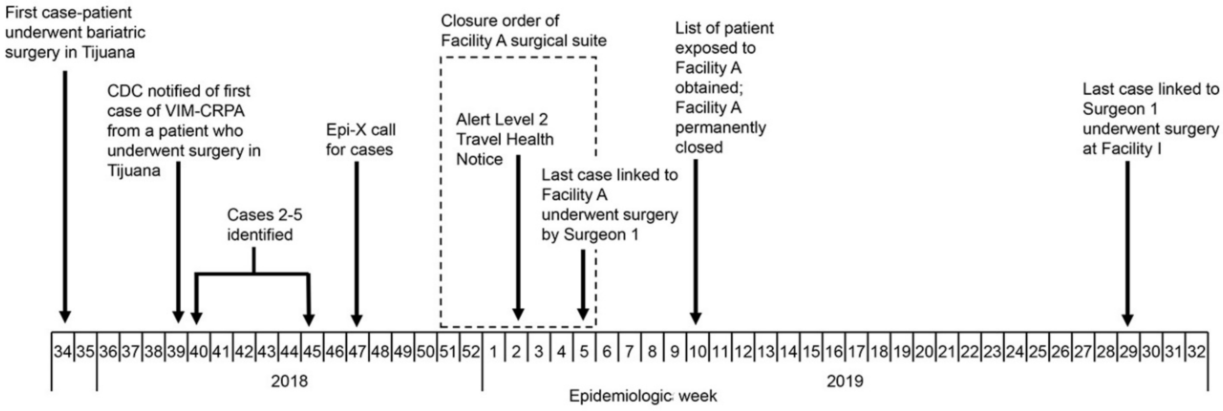

Travel Health Notice Created on January 9, 2019

Alert -Level 2, Practice Enhanced Precautions.

Key Points

• Recently, some US residents returning from Tijuana, Baja California, Mexico, were diagnosed with infections caused by an antibiotic-resistant form of *Pseudomonas aeruginosa* bacteria.

• All of the travelers with this particular infection had an invasive medical procedure performed in Tijuana. Most (but not all) of them had weight-loss surgery. About half of those infected had their surgery done at the Grand View Hospital.

• Based on information provided by the CDC, the Mexican government has closed the Grand View Hospital until further notice.

• CDC recommends that travelers to Tijuana, Mexico, not have surgery at the Grand View Hospital until the Mexican government can confirm that the drug-resistant form of *Pseudomonas aeruginosa* bacteria is no longer there.

What is drug-resistant *Pseudomonas*?

Pseudomonas is a kind of bacteria found widely in the environment. The most common type of *Pseudomonas* that infects humans is called *Pseudomonas aeruginosa*. *Pseudomonas* infections of the blood, lungs (pneumonia), and after surgery can lead to severe illness and death.

Unfortunately, bacteria (including *Pseudomonas*) are becoming more resistant to antibiotics. Infections with bacteria that are resistant to antibiotics are much harder to treat. Bacteria that cause infections that doctors cannot treat easily with antibiotics are called drug-resistant. Drug-resistant *Pseudomonas* bacteria do not respond to most commonly available antibiotics.

What is the current situation?

CDC has received reports of serious drug-resistant *Pseudomonas aeruginosa* infections in US residents who had invasive medical procedures (primarily weight-loss surgery) in Tijuana, Mexico. About half of those infected with these bacteria had surgery at Grand View Hospital, Tijuana. The others became infected after surgery at other hospitals and clinics. Infections caused by this particular drug-resistant *Pseudomonas* are rare in the United States and difficult to treat.

What can travelers to Mexico do to prevent drug-resistant infections?

CDC recommends that travelers to Mexico not have surgery (including weight-loss surgery) at Grand View Hospital in Tijuana, until the Mexican government can confirm that the drug-resistant form of *Pseudomonas aeruginosa* bacteria is no longer there.

Additional information and advice for US residents planning to travel abroad for medical care:

• Some people who have traveled for medical care to countries outside the United States have been infected by hard-to-treat antibiotic-resistant strains of bacteria not commonly seen in the United States.

• Medical and surgical procedures done anywhere (even in the United States) carry some risk and can result in complications.

• See a travel medicine specialist in the United States at least a month before your trip. Travel medicine specialists can provide you with the guidance, vaccines, and medicines you may need for your travel.

• Ask your doctor if you are healthy enough to travel abroad for medical or surgical procedures.

• Research the health care provider who will perform your procedure, as well as the clinic or hospital where you will be receiving care. Be aware that standards for providers and clinics abroad may be different from those in the United States.

• Look for clinics and hospitals accredited by international organizations. Remember that using an internationally accredited facility is not a guarantee that your medical care will be free of complications.

• Ask the clinic or hospital to provide you with copies of all of your medical records. If possible, these records should be in English. Bring them with you to any follow-up appointments you have.

• Anytime you travel outside the country, consider the health and safety concerns at your destination. Also consider the additional risks posed by traveling after surgery:

o Any prolonged travel after Surgery increases your risk of developing blood clots in your legs. Avoid traveling for at least 10 days after surgery on your chest or abdomen (belly). The American Society of Plastic Surgeons recommends that patients wait to fly at least 7–10 days after having cosmetic procedures on the face or after laser treatments.

o Consider the risks of participating in typical vacation activities after surgery. Avoid sunbathing, drinking alcohol, swimming, taking long tours, or participating in strenuous activities or exercise.

If you get sick during or after travel

If you think you have an infection or other complication, seek medical care immediately. Regardless of where you received care, tell your health care provider about your travel and any medical care or surgery you had abroad.

What can clinicians do?

• US health care providers should be vigilant for the possibility of resistant infections occurring in patients who have traveled abroad for medical procedures. Take measures to control the spread of multidrug-resistant organisms in the United States.

• Providers caring for patients with a history of invasive procedures in Mexico should be aware of the potential for infections caused by resistant pathogens. The pathogen implicated in the current cluster of infections is carbapenem-resistant *P. aeruginosa* (CRPA). The resistance mechanism is a metallo-β-lactamase encoded by a mobile genetic element known as the Verona integron.

• CRPA are drug-resistant and difficult to treat, requiring protracted and complex antibacterial drug combinations and courses. Consult with an infectious disease specialist.

• When caring for patients who have a history of having undergone invasive procedures in Mexico, obtain cultures, perform antimicrobial susceptibility testing to guide treatment, and test any carbapenem-resistant bacteria for Verona integron and other plasmid-mediated carbapenemases. Report any CRPA surgical site infections in patients who had invasive procedures in Mexico to your local or state health department.

• When admitting patients who have a history of overnight stays in health care facilities outside the United States, consider performing rectal screening for carbapenemase-producing organisms. This recommendation applies to patients hospitalized outside the United States at any time during the 6 months before their US-based hospital admission.

o Consider placing such patients in isolation and contact precautions while awaiting screening results.

• Mechanism testing for carbapenem-resistant bacteria and rectal screening for carbapenemases are available free of charge via the Antibiotic Resistance Laboratory Network (https://www.cdc.gov/drugresistance/solutions-initiative/ar-lab-network.html), which can be accessed through state health department health care-associated infections programs (https://www.cdc.gov/hai/state-based/index.html).

Traveler Information

• Medical Tourism

Clinician Information

• Medical Tourism in *CDC Health Information for International Travel* ("Yellow Book")

Survey

Form Approved

OMB Control No.: 0920-1253

Public reporting burden of this collection of information is estimated to average 20 minutes per response, including the time for reviewing instructions, searching existing data sources, gathering and maintaining the data needed, and completing and reviewing the collection of information. An agency may not conduct or sponsor, and a person is not required to respond to a collection of information unless

it displays a currently valid OMB Control Number. Send comments regarding this burden estimate or any other aspect of this collection of information, including suggestions for reducing this burden to CDC/ATSDR Reports Clearance Officer, 1600 Clifton Road NE, MS D-74, Atlanta, Georgia 30333; ATTN: PRA 0920-1253

Section 1: Introduction - Notification

Part A

Good morning/afternoon, Mr./Mrs. ____________________,

My name is ______________________. I am calling from the Centers for Disease Control and Prevention, on behalf of _________State Health Department. I am calling because Weight Loss Agents reported to us that you recently had surgery in Tijuana, Mexico. (pause) CDC wants to ensure that you received some important health messages regarding your surgery and potential risks to your health. Do you have a few minutes to speak with me now?

If YES: Continue to Part B

If NO, STOP and collect information: Is there a time that would be better for you?

Day/date____________________

Time___________

Call back number __________

Part B

Have you received an email or other communication from Weight Loss Agents about potential infections following surgery at Grand View Hospital?

If YES: Skip to section 2

If NO: Continue

Thank you for taking the time to talk to me today. CDC has received multiple reports of infections following weight loss surgery at Grand View Hospital in Tijuana, Baja California, Mexico. These infections were caused by a certain type of bacteria that is resistant to most antibiotics. That means bacteria have developed the ability to defeat drugs designed to kill them. When Mexican officials investigated possible sources of these infections, they discovered cleaning and disinfecting medical devices was performed improperly.

If you are hospitalized between now and December 31, 2019, please tell your doctor, at the time of admission, that you had surgery at Grand View Hospital and were contacted by the CDC. We will send you a letter that you should share with your doctor if you are admitted to a hospital explaining the risk.

Do you have any questions?

Because of the practices found at the hospital, there is also a very low risk that you may have been exposed to other germs, including HIV, hepatitis B and C viruses. We are not aware of any HIV, hepatitis B, or hepatitis C infections at this facility, but to be safe, we recommend that you also talk to your doctor and get tested for these 3 infections.

I understand that this information is alarming and may be frightening. Do you have any questions?

Skip to Section 3

Section 2 (Only if Yes to Section 1, Part B)

The notification described a risk of infection with antibiotic resistant bacteria. That means bacteria have developed the ability to defeat drugs designed to kill them. If you are hospitalized between now and December 31, 2019, please tell your doctor, at the time of admission, that you had surgery at Grand View Hospital and were contacted by the CDC. We will send you a letter that you should share with your doctor if you are admitted to a hospital explaining the risk.

Do you have any questions about the information in the notification?

Because of the practices found at the hospital, the notification also included information about the risk of other germs including HIV, hepatitis B and C viruses. We are not aware of any HIV, hepatitis B, or hepatitis C infections at this facility, but to be safe, we recommend that you also talk to your doctor and get tested for these infections.

Do you have any questions? (go to section 3, survey)

Section 3 - Survey

I would like to ask a few additional questions about you, your recent surgery, and your health. This will only take about 20–25 minutes. Information you provide will help us better understand the risks of infection and could help prevent others from getting sick. Answering these questions is completely voluntary. You may choose to skip questions or end the interview for any reason at any point. Everything you tell me will be kept confidential and will not be shared outside the public health

investigation group. We may publish information related to this investigation in a medical journal but we cannot use your name or personal information.

Is it okay if I ask you these questions? Yes/No

• If no – That's okay, could we schedule another time for me to ask these questions, or would you prefer not to answer these questions

(1) Patient ID

(2) What is your age? (number)

(3) What is your gender? Female/Male

(4) Would you describe yourself as Hispanic or Latino/a? Yes/No/Refused or Unknown

(5) How would you describe your racial background? White/Black or African American/Asian/American Indian or Alaska Native/Native Hawaiian or Other Pacific Islander/Refused

(6) What is your occupation? (free text)

Now, I am going to ask some questions about your surgery at Grand View Hospital.

(7) What type of surgery did you have? If you had more than one surgery, please tell me about all surgeries that you had at Grand View Hospital. Check box, open, laparoscopic, c gastric sleeve surgery, gall bladder removal, revision surgery, gastric bypass, intragastric balloon, unknown, other *(free text)* check all apply.

(8) What was the date(s) of surgery(s) in Mexico?

(9) What is the name of the person(s) who performed your surgeries in Mexico (?) Mario Almanza, Marcelo Hernandez, Rafael Camberos, Alberto Michel, Rafael Michel, David Vazquez, Galileo Villarreal, Francisco Vavalza, Other (free text), Other, Unknown or don't remember

(10) Have you been diagnosed with an infection that might be related to your surgery at Grand View Hospital? Y/N

• **(10 yes a) If yes** – What type of infection were you diagnosed with? Infected surgical wound, abscess (which is a pus-filled sac or boil), sepsis, blood stream infection, other (free text)

(10 yes b) A culture is when a body site like a wound is swabbed or body fluids like urine or blood are collected to look for a bacteria. For this infection, was a culture collected?

▪ (**10 yes c)** If yes - Which body site(s) was the culture taken? (free text) Medical facility where collected? (free text) Dates collected? (free text) Did any bacteria grow from the culture? Yes/No Do you know the name of the bacteria that grew from the culture? (free text)

• **(10 no a)** If no - Have you had any of the following signs of infection since your surgery?

• Warmth, redness, or swelling at incision site? Y/N

• Fluid draining from incision site? Y/N

• Pus at incision site? Y/N

• If yes to any: Did you have a fever at the same time? Y/N

• If yes to any: Did you seek medical care for this problem? Y/N

• If no**:** If you have not sought medical care, please consider doing so. If you have an infection, it is important to be treated promptly. Let your physician know about this investigation and the concern for possible infection. You can also have the physician call CDC (add number) or the local health department.

Now I'd like to ask some questions about recent hospitalizations you might have had.

(11) In the year prior to your surgery at Grand View Hospital, how many times were you admitted to a hospital for one or more nights? 0/1/2/3 or more

(12) Since returning to the United States after your surgery, have you been admitted to a hospital for one or more nights? Y/N

• **(12 yes a)** What was the name of the hospital where you were most recently admitted? (free text) What were the dates of your hospitalization? What was the reason for this hospitalization? Where were you discharged to? (Home/skilled nursing facility/long-term acute care facility/inpatient rehabilitation facility)

• Have you had any additional hospitalizations since returning to the US? Y/N What was the name of the hospital? (free text) What were the dates of your hospitalization? (free text) What was the reason for this hospitalization? (free text) Where were you discharged to? (Home/skilled nursing facility/long-term acute care facility/inpatient rehabilitation facility)

•

Last, I am going to ask you some general questions about your experience. Some of these questions may be sensitive. Remember, you do not have to answer any question. If you want to skip a question, just ask to move to the next one.

(13) Which of the following factors contributed to your decision to have surgery in Tijuana? Cost, Recommendations from family/friends, Did not meet weight qualifications for surgery in US, Short wait time, Fewer preparation requirements, Other (free text)

(14) Prior to surgery, did you have concerns about the quality of care you would receive by the physician or the surgical center that you chose? Yes/No/Declined/Don't know

(15) Before having your surgery in Tijuana, were you aware of any advisories, warnings, or media stories regarding Grand View Hospital in Tijuana? Y/N

• **(15 yes a)** (If yes, please tell us where you received information? State health department alert/Local news story (TV/newspaper)/National news story (TV/newspaper)/CDC Travel Alert/Social media/Online article about outbreak at Grand View/Online article about litigation involving surgeon at Grand View/ Other (free text)

• **(15 yes b)** If you were aware of any advisories, warnings or media stories, above, what factors contributed to your decision to go ahead with surgery in Mexico? cost, already paid, unable to receive refund for deposit, already booked travel, short wait time, received reassurances from travel agency, received reassurances from hospital , received reassurances from previous patients, had surgery there in the past/other

(16) Is there any additional information would have been useful in making your decision to have surgery in Mexico? (Free text)

(17) Where would you usually get information about travel alerts or warnings? State health department website/CDC website/Travel agency website/Facebook/Other social media (specify)/Other/I don't know where to look for information

(18) Have you spoken with your doctor about the recommendation to consider getting tested for hepatitis and HIV? Y/N

• **(18 yes a)** If yes - did you undergo testing? (you do not have to share results)

• **(18 no b)** If no – what is the primary reason you have not spoken with your provider? did not know about recommendation/afraid of result/have not had time/no insurance to cover testing/other (free text)

(19) Is there anything else you would like us to know? (free text)

Thank you very much for talking the time to talk to me today. If you think of something else you want to share, you can call [number] or you can email [email address of box].
